# Supplementary material for: Molecular Methods for the Simultaneous Detection of Tomato Fruit Blotch Virus and Identification of Tomato Russet Mite, a New Potential Virus–Vector System Threatening Solanaceous Crops Worldwide
Source: Viruses. 2024 May 18;16(5):806. doi: 10.3390/v16050806 (PMC11126101; doi:10.3390/v16050806)
Supplement: Supplementary file 1 [file viruses-16-00806-s001.zip › viruses-3012183-supplementary.pdf]

Supplementary Materials

Table S1 – List of the samples used for assessed analytical specificity as inclusivity and exclusivity. For each a result positive (+) or negative (–) was reported. For the duplex assay the target or targets that reacted were reported in the brackets. NT – not tested.

		Real-time RT-PCR			*ddRT-PCR*	
	sample ID	ToFBV	TRM	duplex	ToFBV	TRM
inclusivity results	92	+	-	+ (ToFBV)	+	-
	94	+	-	+ (ToFBV)	+	-
	95	+	-	+ (ToFBV)	+	-
	96	+	-	+ (ToFBV)	+	-
	97	+	-	+ (ToFBV)	+	-
	98	+	-	+ (ToFBV)	+	-
	99	+	-	+ (ToFBV)	+	-
	1	+	+	+ (ToFBV) + (TRM)	+	+
	2	+	+	+ (ToFBV) + (TRM)	+	+
	4	+	+	+ (ToFBV) + (TRM)	+	+
	5	+	+	+ (ToFBV) + (TRM)	+	+
	104	+	-	+ (ToFBV)	+	-
	1B	+	+	+ (ToFBV) + (TRM)	+	+
	1C	+	+	+ (ToFBV) + (TRM)	+	+
	TRM b from sample 1B	+	+	+ (ToFBV) + (TRM)	+	+
	TRM b from sample 1C	+	+	+ (ToFBV) + (TRM)	+	+
	TRM from *S. nigrum*	+	+	+ (ToFBV) + (TRM)	+	+
	TRM from *S. nigrum*	+	+	+ (ToFBV) + (TRM)	+	+
exclusivity results	*S. lycopersicum*	-	NT	-	-	NT
	S. nigrum	-	NT	-	-	NT
	C. arvensis	-	NT	-	-	NT
	CEVd	-	NT	-	-	NT
	CLVd	-	NT	-	-	NT
	CMV	-	NT	-	-	NT
	INSV	-	NT	-	-	NT
	PCFVd	-	NT	-	-	NT
	PePMV	-	NT	-	-	NT
	PmoV	-	NT	-	-	NT
	PSTVd	-	NT	-	-	NT
	PVY	-	NT	-	-	NT
	TASVd	-	NT	-	-	NT
	TCDVd	-	NT	-	-	NT
	TICV	-	NT	-	-	NT
	TMV	-	NT	-	-	NT
	ToBRFV	-	NT	-	-	NT
	ToCV	-	NT	-	-	NT
	ToLCNDV	-	NT	-	-	NT
	ToMMV	-	NT	-	-	NT
	ToMV	-	NT	-	-	NT
	TPMVd	-	NT	-	-	NT
	A. tosichella	NT	-	-	NT	-
	P. adalius	NT	-	-	NT	-
	B. tabaci	NT	-	-	NT	-
